# Parental Perceptions of the Impact of a Child’s Complex Chronic Condition: A Validation Study of the Impact on Family Scale

**DOI:** 10.3390/ijerph21050642

**Published:** 2024-05-17

**Authors:** Sandra Portela Alves, Ana Carolina Braz, Luís Graça, Anne Marie Fontaine

**Affiliations:** 1 Faculty of Psychology and Educational Sciences, University of Porto, 4200-135 Porto, Portugal; fontaine@fpce.up.pt; 2 Independent Researcher, 3030-326 Coimbra, Portugal; anacarolbraz@gmail.com; 3 Superior Health School, Polytechnic University of Viana do Castelo, 4900-347 Viana do Castelo, Portugal; luisgraca@ess.ipvc.pt

**Keywords:** pediatric complex chronic illness, impact on family, satisfaction with life, parents, validation study

## Abstract

The diagnosis of a child’s complex chronic illness may impact family relationships and cohesion. The Impact on Family Scale (IFS) is an instrument used to assess the parental perception of the effects of children’s chronic illness on family life. With a sample of 110 mothers and fathers between the ages of 29 and 50 who have a child with a complex chronic illness, we examine evidence of the validity of the IFS for use in Portugal within this specific family configuration, (1) comparing its factor structure with the original one; (2) assessing its reliability; and (3) evaluating its relationship with life satisfaction and family cohesion/acceptance. As expected, CFA analysis showed that IFS is a one-factor reliable instrument with 12 items (Cronbach’s alpha = 0.910), which are negatively correlated with satisfaction with life (r = −0.229, *p* = 0.016) and positively correlated with family acceptance and cohesion (r = 0.363; *p* < 0.001). The results support the validity of the IFS in families with children and adolescents with a complex chronic illness. The implications of the use of this instrument for research and professional practice is analyzed.

## 1. Introduction

A chronic disease that extends over time or requires continued hospitalization interferes significantly with daily life and family dynamics. Each individual member of the family and the family system as a whole develop very specific strategies to improve the adaptation to and integration of the disease that threatens the child’s life, seeking to achieve a rebalance that favors recovery from this moment of crisis [1].

Complex chronic illness in children is generally defined as a condition that lasts for at least 12 months, that affects several systems or an organ in a way that requires specialized pediatric care and some period of hospitalization [2]. It is severe enough to pose limitations to the activities of daily living of the child and/or their family.

As mentioned by Kazak [3], the experience of this type of illness impacts the family significantly, constituting a stressful situation. The family is confronted with new demands, changes at various levels, and constant readaptations, sometimes over several years. Such adaptations are particularly demanding for mothers and fathers, although the various systems of an interactive nature in which the child and family are inserted contribute significantly to their adjustment, seeking a balance between risk and protection factors [4,5]. It can be difficult for fathers and mothers to shift the focus from the centrality of the need to care for the child to self-care as caregivers [6]. Also, siblings experience changes in their routines and family roles, and intense feelings arise, particularly in periods of exacerbation of the disease or during moments of hospitalization of the sibling [7]. In this context, in which the family deals with a crisis, it seems important to assess the parent’s perception of burden, in the sense of the negative effects that result from providing care to a child with a prolonged disability and/or chronic illness [8]. Indeed, attitudes of mothers and fathers inevitably influence the quality of family functioning, its cohesion, and the way in which each member of the family adapts to this experience, as well as the relationship established with others, for example, professionals who take care of the child [9]. The parental perception of the quality of family functioning is an important predictor regarding the process of adaptation to complex chronic pediatric disease, which is related to the parents’ satisfaction with life (SWL).

### 1.1. Assessment of the Impact of Complex Chronic Illness on Family

Measures to assess the family impact of a child’s chronic complex illness are needed to be able to manage intervention with this specific population, beyond hospital contexts. There are scales that measure parental stress—e.g., [10,11]—but not the family impact; scales that measure the negative and positive implications that arise from the child’s disability—e.g., [12]—or the impact of hospitalization are therefore not sensitive to outpatient and home contexts [13]. The existence of few assessment scales in this area itself reveals the relevance of having a reliable scale with which to assess the impact of complex chronic disease on families in a wide range of contexts.

The Impact on Family Scale (IFS) assesses the family impact of a child’s chronic disease. The original scale was designed by Stein and Riessman [14] and is one of the most used in international studies. This scale, composed of 24 items, is organized into four factors: financial burden (economic consequences for the family); familial/social impact (disruption of social interaction); personal strain (tension or psychological overload experienced by the primary caregiver); and mastery (coping strategies used by the family). The adoption of the IFS in several countries (e.g., France, Germany, Turkey) confirmed its multidimensional structure, although the mastery dimension is less reliable and some items of the financial dimension are less suitable in some contexts [15,16], as cited in Albuquerque et al. [17]. Stein and Jessop [18] suggested a reduction in the number of items in the original version to 15, which only covered the dimensions Family/Social Impact and Personal Strain, with a one-dimensional structure. This shorter version of the scale has been widely used [15,19,20,21,22,23,24], and it was adapted in Portugal by Albuquerque et al. [17]. Although the first results of this Portuguese adaptation support the unidimensional structure of the scale, they only pertain to parental burden in very specific contexts, such as the hospital, with parents being informed of the diagnosis of their child’s disabilities, uropathies, and cardiopathies just before or after birth.

There are few studies with the short version of IFS that apply beyond this kind of sample. Therefore, validity studies of IFS with patients covering a wider range of chronic complex illnesses and ages, including older children and adolescents whose diagnosis has been known for many years, are needed. Suggestions from the authors of the first IFS Portuguese version are to expand the representativeness of the sample and situations [17]. Moreover, the construct validity of the instrument needs to be better assessed.

### 1.2. Complex Chronic Illness, Life Satisfaction, and Family Cohesion/Acceptation

To estimate construct validity, we aim to observe the relation between IFS results and satisfaction of life, as well as two interrelated aspects, acceptance and cohesion, central for the quality of family functioning.

Mothers and fathers assume a dual role between the exercise of parenthood and the task of being an informal caregiver. Integrating the demands arising from this specific caregiving task in a context of complex chronic illness and parental daily life can represent an added difficulty. The literature points out that greater difficulties in this process seem to be associated with lower parental satisfaction in relation to the performance of the parental role [25]. When the task of caring is demanding and poses constant challenges to family dynamics, parents may experience greater difficulty in taking care of their own physical and emotional health [26], which can impact their satisfaction with life.

On one hand, if satisfaction with life as conceptualized by Diener [27] represents a global synthesis based on the multiple perceptions of satisfaction in different areas of life, the perception of greater burden on fathers and mothers when coping with their child’s chronic disease may reduce their perception of satisfaction with life. On the other hand, over the years, parents who deal with their child’s complex chronic disease can also develop cognitive and emotional coping strategies that reduce daily stress and improve acceptance of the situation [28]. Acceptance can be defined as the attempt to assign meaning to the child’s disease, striking a balance between the parent’s values, beliefs, and goals, and control over emotional reactions to what they are experiencing [29,30]. Acceptance is an inner attitude that seems to be related to positive affects. Moreover, Mendes et al. [31] found that family cohesion was associated with the adaptation outcomes for both children and their parents via parental mutuality and the perception of family life difficulty: when parents reported higher levels of family cohesion, they also reported lower levels of family life difficulty and higher levels of parental mutuality. Family cohesion increases its members’ acceptance of the disease constrains, supports management behaviors associated with the establishment of family routines related to treatments, and increases compliance with therapy [32,33]. Therefore, these constructs are associated with each other. In light of these factors, a positive association between results of the IFS and family acceptance/cohesion is expected, but we expect a negative association between results of the IFS and satisfaction with life.

It is crucial to assess how parents cope with the diagnosis of a child’s chronic illness and its impact on the family. A methodological study that aims to validate and adapt a pre-existing instrument that contributes to the consolidation of care protocols is needed. Regarding the importance of this, the present study aims to evaluate the psychometric properties of the Portuguese version of the short form of the Impact on Family Scale (IFS-PT, 15 items), in a sample of parents of children and adolescents diagnosed with broad-ranging complex chronic diseases, assessing (a) the factorial validity of the IFS-PT, (b) evidence relating to its reliability, and (c) its construct validity.

## 2. Materials and Methods

### 2.1. Participants

The sample of this study included 110 parents, comprising 57 mothers aged between 29 and 50 years (M = 40.33, SD = 4.241), and 53 fathers aged between 37 and 42 years (M = 39.50, SD = 3.536). Regarding the educational level, 57.9% of mothers and 47.2% of fathers had secondary or higher education. Inclusion criteria included being a father or mother of a child aged between 3 and 17 years, with a complex chronic disease diagnosed at least six months before the data collection period, and being able to read and understand the questionnaires. In this sample, the minimum period living with the verified disease situation, after the diagnosis, was one year, and the maximum was nine years. Diagnoses included several conditions, including neurological, cardiovascular, oncologic, genetic, renal, and metabolic diseases, chromosomal disorders, cerebral palsy, and brain malformations. Mothers were the main caregivers in 97.5% of the cases. Concerning household composition, 54.4% of families had at least two children under the age of 18, while 14% had between three and five children under the age of 18. Families were recruited through intervention services designed for children with complex chronic diseases in different health facilities and programs. The sample size was estimated in accordance with the criterion in [34] of a 5:1 subject–item ratio, for confirmatory factor analyses (CFAs). Data collection occurred from 2017 to 2018. This study is part of a larger project on family intervention in the case of children with complex chronic illness.

For this study, we used convenience sampling procedures to recruit participants. We invited participants to take part in our study through initially contacting the directors of institutions (the institutions contacted were schools, through their special education teams; cerebral palsy associations; early intervention teams; and non-governmental organizations that provide support in these situations of complex chronic disease), where children are usually cared and accompanied. The family monitoring technician firstly approached each family about their participation in the study, explaining the general objectives. After consenting to being contacted by the researchers, the first round of face-to-face contact took place with each mother and father to gather information.

The study was approved by the Ethics Committee of the Faculty of Psychology and Educational Sciences of the University of Porto, Portugal (FPCEUP) (protocol number 7-9/2015). We explained the aims of the research project and ethical protections for the study participants (for example, anonymous and voluntary participation and the possibility to withdraw from the study without any consequences) before initiating data collection. Participants were then invited to sign informed consent forms so that we could proceed with the study. All participants used the proposed instruments.

### 2.2. Instruments

A questionnaire was used to collect sociodemographic data on the participants (age, gender and education) and their family characteristics (household members and characteristics of the children with complex chronic illnesses (age, gender, and history related to their diagnosis)).

To assess the negative impact of the complex chronic disease on the family, conceptualized in terms of restrictions, losses, suffering, and subjective emotional tension, we used the short version of the Impact on Family Scale—IFS-PT—a 15-items scale, answered on a 4-point Likert scale on the degree to which each statement applies to the parent (from 1 = Strongly Disagree to 4 = Strongly Agree) [18]. Studies achieved with this short scale suggest the use of a single burden perception dimension, which includes items from the Familial/Social Impact and Personal Strain dimensions of the longer original version of the Impact on Family Scale (e.g., “we spend less time with family and friends due ’o my son’s health problem”; “No one understands the burden I have”). The first Portuguese study using this short version with parents of babies, in hospital settings at birth, considered it adequate for use, both in clinical practice and in research [17]. Indeed, it showed good internal consistency (Cronbach’s alpha = 0.91), temporal stability (r = 0.80, *p* < 0.001), acceptable results in terms of factorial validity, as well as little evidence of convergent and discriminant validities. Higher scores, which correspond to the sum of the items, indicate a perception of there being a greater impact of the baby’s health condition on the family.

Satisfaction with life was measured using the Satisfaction with Life Scale (SWLS) [35] adapted to European Portuguese standards by Simões [36]. This is a five-item unidimensional instrument that asks people about their feelings and attitudes regarding the current moment of their lives (e.g., “In most ways, my life is close to my ideal”), and is widely used to measure global life satisfaction. Items are rated on a 5-point Likert scale from totally disagree (1) to totally agree (5). Cronbach’s alpha value observed in the current study was 0.75. The use of this scale in a lot of cultures confirms its unidimensionality and reliability [36,37,38,39,40].

As family cohesion and acceptance are highly associated, they were assessed using a single dimension of the Positive Contributions Scale (strength and family closeness), from the Kansas Inventory of Parental Perceptions—KIPP [41]—adapted to European Portuguese standards by Fonseca and colleagues [42], based on cognitive adaptation theory and the family theory of stress and coping. This scale assesses the perception of parents who have a child with disabilities and special needs and focuses on positive dimensions and successful coping (e.g., “The presence of my child makes us more in charge of ourselves as a family”; “Because of my child I am more sensitive to family issues”), and is answered on a 4-point Likert scale (from 1 = Strongly Disagree to 4 = Strongly Agree). Cronbach’s alpha value observed in the current study was 0.69, close to 0.70, which is considered acceptable [34].

### 2.3. Data Analysis

Values of skewness (sk), and kurtosis (ku) were analyzed to judge the distributional properties of each item. In the context of factor analysis, as absolute value of asymmetry lower than 2, and absolute value of kurtosis lower than 3, indicators of a normal distribution [43] are needed to use parametric statistical procedures, such as confirmatory factor analysis (CFA) [44]. The absence of outliers and multicollinearity are also required for CFA. The presence of outliers is checked through Mahalanobis distance and multicollinearity through inter-item correlations. We conducted CFA, using Amos 18.0 [45], and compared its results with the previously established unidimensional factor structure recommended by the authors of the short version of the IFS [18]. Previously, multivariate outliers were detected through the Mahalanobis distance measure (D2) (*p*1 and *p*2 < 0.001), as was the existence of multicollinearity based on the inter-item correlations. If values are greater than 0.90, items must be excluded, as recommended by Marôco [34]. The overall fit of the hypothesized model was evaluated based on Kline [46] and Schweizer [47] criteria, which recommended, for little samples with a normal distribution, that the χ^2^ test cannot be significant, the Bentler comparative fit index (CFI) should be > 0.90 [48], the root mean square error of approximation (RMSEA) should be < 0.08 [45] and the standardized root mean square residual should be (SRMR) < 0.10 [48]. We also evaluated local fit based on standardized factor loadings (>0.50) and the individual item reliability coefficients (>0.25), which led to the confirmation of the factor validity of the model [34].

The construct validity of the IFS-PT was assessed through its correlation with the SWLS and the dimensions of cohesion/acceptance of the KIPP. A negative correlation was expected between family burden, assessed by the IFS-PT, and SWL. Considering the experience of the family and the opportunity to develop cognitive and affective strategies to cope with the stress associated with the chronic disease of the child, as proposed by Cici and Özdemir [28], a positive correlation was expected between IFS-PT and cohesion/acceptance.

## 3. Results

### 3.1. Evidence of Construct Validity Based on Internal Structure

After assessing the distributional properties of the sample data, all values of skewness and kurtosis for each item were below the critical values defined by Finney and DiStefano [43] in our study (below 2 for both skewness and kurtosis), indicative of a normal distribution, allowing us to use parametric statistical procedures.

We did not find outliers, but some evidence of multicollinearity was observed between item 1, item 7, and item 8. Thus, to avoid suppressor variable problems, the following three items were excluded [34]: item 1 (“Because of the illness, we are not able to travel out of the city”), item 7 (“Sometimes I wonder whether my child should be treated “specially” or the same as a normal child”), and item 8 (“I think about not having more children because of the illness”) [34]. Skewness and kurtosis values for the 12-item scale are available in Table 1.

**Figure 1:**
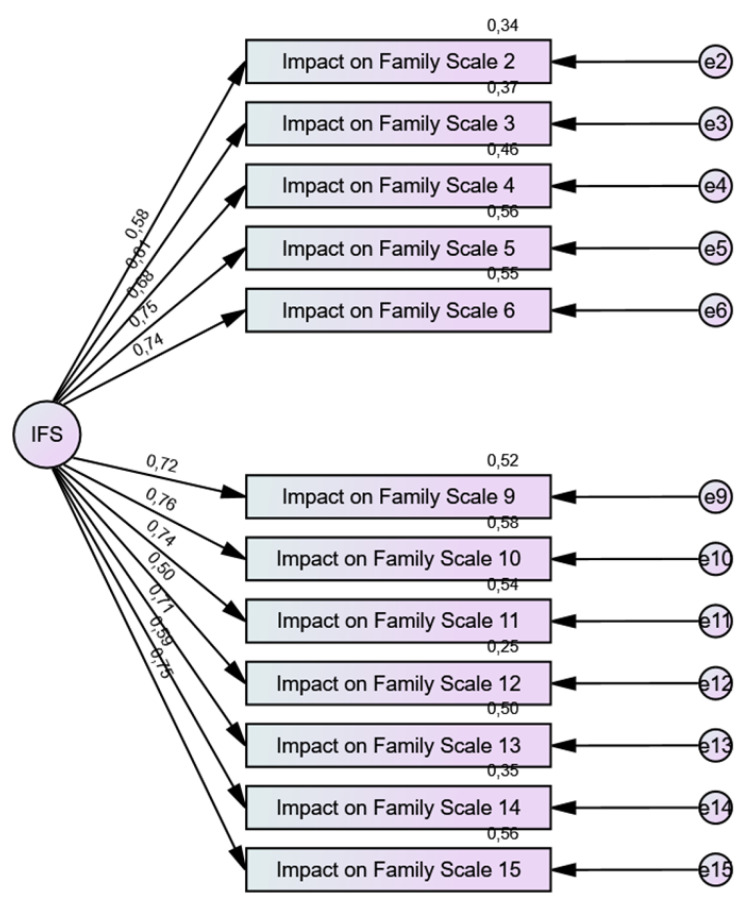
Confirmatory factor analysis of the IFS-PT.

We decided to test the unidimensional structure of the scale reduced to 12 items through confirmatory factor analyses (CFA) (Table 2). We chose not to correlate the errors, keeping the model as parsimonious as possible, given the confirmatory nature of the model. The model fit the data well (Figure 1), with value reaching the assumed standard: χ^2^ is not significant (*p* = 0.165); the Bentler comparative fit index (CFI) = 0.982; the root mean square error of approximation (RMSEA) = 0.041; and the standardized root mean square residual (SRMR) = 0.045. We prioritized theoretical fit; therefore, no changes were made to the model specification. All estimated parameters remained statistically significant (*p* < 0.001). With respect to local fit, standardized factor loadings were all above 0.50, and coefficients for the item’s reliability were all above 0.25. These local values are also indicators of good fit, and, consequently, support the factor validity of the one-factor model [34].

### 3.2. Evidence of Construct Validity Based on Reliability

Cronbach’s alpha for the total 12-item scale was 0.910, confirming its excellent internal consistency.

### 3.3. Evidence of Construct Validity Based on Criterium Validity

We computed the total score of the 12-item IFS-PT and correlated it with the total scores of the SWLS and with the Family Cohesion/Acceptance dimension. We found a negative moderated correlation between the IFS-PT and satisfaction with life (r = −0.229; *p* = 0.016), and a positive moderate correlation between IFS-PT and the cohesion/acceptance dimension (r = 0.363; *p* < 0.001) [34].

## 4. Discussion

In studies carried out in other cultures, it has been documented that the Impact on Family Scale (IFS-PT) is a valid scale for assessing impact on the family when they experience a chronic pediatric illness in different contexts, and it may be used either in clinical settings or research settings [16,18,24,49,50,51,52,53]. Regarding the cultural invariance of the IFS-PT, [15], using the 24-items version of IFS, we identified three factors (financial burden, familial/social impact, and personal strain) that were useful for cross-cultural comparisons between a U.S. and an Italian sample. However, the financial factor is not stable in other cultures, and we opted, as did Stein and Jessop [18], for the 15-item scale, which Albuquerque also used in the study with babies’ parents [17]. In her study, the psychometric properties of this scale were good, in very specific conditions: in a clinical context, with families who were coping with the recent announcement of their babies’ impairment, and in some complex chronic disease [17]. To use this scale in other contexts, for other diseases and for other age ranges, evidence of validity is needed.

In this study, we aimed to assess the construct validity of the IFS-PT through its factorial structure, using confirmatory factor analysis (CFA), its internal consistency, and criterium validity to confirm the adequacy of the IFS-PT for a sample of parents whose children have a complex chronic disease, taking into account a wide spectrum of diagnoses that including neurological, cardiovascular, oncologic, genetic, renal, and metabolic diseases, chromosomal disorders, cerebral palsy, and brain malformations, and with both children and adolescents (3 to 17 years old). Through the confirmatory factorial analysis conducted with families that have children with complex chronic illnesses and that have been coping with this problem for several years, we obtained a 12-item structure, which confirm the unifactorial structure of the scale and has excellent internal consistency.

We also evaluated the criterium validity of this 12-item Portuguese scale. So far, the population in question indeed seems to meet specific conditions that sustain the correlations observed. The negative moderate correlation between IFS-PT and satisfaction with life (r = −0.229; *p* = 0.016) was expected, as, given the perception of greater burden by fathers and mothers, there is a global perception of lower satisfaction with life. We cannot ignore the fact that these experiences of complex chronic pediatric diseases are frequently associated with a dimension of uncertainty regarding the future, whether in relation to treatments, the prognosis of the disease, or systemic dimensions associated with the socioeconomic situation of the family or even concerns about who will take care of the child if the parents, for some reason, are no longer able to do so. This uncertain context requires constant parental adjustment to everyday demands and constitutes a robust predictor of distress [54], which may impact the parental perception of satisfaction with life.

However, this situation also has a positive impact on the family system. We found a positive moderate correlation between this dimension and the 12-item IFS-PT (r = 0.363; *p* < 0.001). As expected, to deal with a greater perception of burden, the family seeks to develop family cohesion to support the behaviors that favor the family’s adaptation to this experience and that allow it to adapt the child’s care routines [33]. However, the moderate correlation suggests, in this case, acceptance and family cohesion as protective factors. As we indicated before, acceptance seems to be related to positive affection. Despite the crisis and its continuity over time, families and individuals increase their acceptance of the disease in terms of the normalization of some routines, improving the family’s adaptation. Family strategies make it possible to manage the impact of burden on the family unit, increasing family cohesion. Family cohesion has been highlighted as a resilience factor in terms of parental adjustment [55] associated with the implementation of the family’s strategy to deal with the fact that there is a child with a chronic illness [56]. This seems to be coherent with the findings of Moreira et al. [57]. They explained that there is a link between family cohesion and parents’ adjustment: the more the family environment is supportive and caring, the less they tend to negatively evaluate the impact of the child’s chronic illness. As the value of internal consistency of the cohesion/acceptance dimension is relatively low (0.69), the above results should be interpreted with caution.

Nevertheless, it is expected that families’ experience of living with a pediatric chronic complex disease will impact each member, but also the entire family’s dynamics and structure. From a systemic view, the disease dimension, as a moment of crisis experienced by the family, can also constitute an opportunity for the development of family resilience. Although we cannot devalue the uncertainty that these situations entail, both in terms of prognosis and in terms of the management of worsening symptoms that require periods of hospitalization for the child, for example, this situation requires that the family as a whole progressively finds strategies that will allow its members to integrate each other’s different demands, including the target child, leading to a reorganization of the family system [9]. Indeed, parents may develop strategies over time that allow them to adaptively manage social and family impacts on personal distress.

Nevertheless, this study also has some limitations. First, this study analyzed the opinions of fathers and mothers as individuals. More studies are needed that consider the parental couple as a unit of analysis. Second, in our sample, all families had two or more children. Although this opens up the avenue for a systemic conception of family experiences, families with only one child and other forms of parenting were not represented. From a systemic point of view, more studies are also needed to better explore family strategies for dealing with the disease process. Finally, our data are transversal. Longitudinal studies are needed to better understand the adaptation process that families develop throughout this experience, and, consequently, its impacts over time.

## 5. Conclusions

Having access to a valid and reliable short version of the Impact on Family Scale (IFS-PT) is important for both researchers and professionals dealing with families who have a child with a complex chronic disease. This study presents evidence for the construct validity of the short version of the IFS-PT with a sample of 110 Portuguese mothers and fathers between the ages of 29 and 50, with children aged between 3 and 17 years, with a complex chronic disease diagnosed at least six months before the data collection period. In our sample, we had families dealing with the disease process for different periods, with a maximum of nine years after diagnosis. The results of a confirmatory factor analysis, which confirm the scale’s one-factor structure and its reliability, maintain the validity of a 12-item version of the Portuguese IFS-PT scale. Construct validity based on its relationship with satisfaction with life (SWL) and family cohesion/acceptance reinforce the construct validity of the 12-item IFS-PT.

A greater consequence of the validation of this scale is the adequacy of its use to assess the impact of chronic complex diseases on the family, considering a wide spectrum of chronic complex diseases in children and adolescents, whenever the time of the diagnosis (one year to several years) or the setting of the assessment (a hospital or another context). Apart from its obvious importance for assessing the family impact of a child’s chronic illness in any research, this refined short version of the IFS may also be useful for ensuring a more responsible application of the scale in the clinical context. In fact, the use of this scale will guarantee the provision of better psychological health care, given the reliability and validity of the information it enables to be collected in a wide range of clinical subpopulations.

In addition, the negative moderate correlation between the IFS-PT and SWL scale and the positive moderate correlation between the IFS-PT and the cohesion/acceptance dimension have some implications for clinical intervention. It seems important to promote interventions that help in developing family skills to deal effectively with the challenges that arise from living with someone with a complex chronic disease, regardless of whether or not they experience psychopathological symptoms. Interventions will support families to cope with stress and uncertainty, and to improve their cohesion and adaptation, and therefore the family resilience. Beyond their implications for research and clinical practice, the data may also, in the future, sustain interventions focused on family-protective factors, including variables such as acceptance and cohesion.

## Figures and Tables

**Table 1:** Descriptive statistics for the 12- item IFS-PT (N = 110).

		Minimum	Maximum	Mean	Standard Deviation	Skewness		Kurtosis	
item							SE		SE
IFS2		1	3	1.70	0.773	0.578	0.230	−1.094	0.457
IFS3		1	4	1.63	0.844	1.266	0.230	0.877	0.457
IFS4		1	4	2.25	1.070	0.250	0.230	−1.212	0.457
IFS5		1	4	2.04	1.022	0.504	0.230	−0.981	0.457
IFS6		1	4	1.85	0.985	0.827	0.230	−0.489	0.457
IFS9		1	4	1.93	0.945	0.612	0.230	−0.718	0.457
IFS10		1	4	2.21	1.050	0.150	0.230	−1.317	0.457
IFS11		1	4	2.32	0.967	0.002	0.230	−1.067	0.457
IFS12		1	4	2.55	0.992	−0.211	0.230	−0.981	0.457
IFS13		1	4	1.81	0.904	0.997	0.230	0.253	0.457
IFS14		1	4	2.04	1.004	0.370	0.230	−1.182	0.457
IFS15		1	4	2.33	1.093	0.088	0.230	−1.339	0.457

**Table 2:** Evolution of the 15-item and 12-item IFS-PT model.

	χ^2^ (*p* Value)	RMSEA	CFI
Model with 15 items	0.002	0.067	0.937
Model with 12 items	0.165	0.041	0.982

## Data Availability

The anonymized data of this study may be provided on request to the first author.
